# What Factors Influence Obesity in Spain? A Multivariate Analysis of Sociodemographic, Nutritional, and Lifestyle Factors Affecting Body Mass Index in the Spanish Population

**DOI:** 10.3390/healthcare13040386

**Published:** 2025-02-11

**Authors:** Elena Sandri, Michela Piredda, Marco Sguanci, Stefano Mancin

**Affiliations:** 1 Faculty of Medicine and Health Sciences, Catholic University of Valencia San Vicente Mártir, c/Quevedo, 2, 46001 Valencia, Spain; 2 Doctoral School, Catholic University of Valencia San Vicente Mártir, c/Quevedo, 2, 46001 Valencia, Spain; 3 Research Unit Nursing Science, Department of Medicine and Surgery, Campus Bio-Medico di Roma University, Via Alvaro del Portillo, 21, 00128 Rome, Italy; marco.sguanci@unicampus.it; 4 IRCCS Humanitas Research Hospital, Via Manzoni, 56, 20089 Rozzano, Milan, Italy; stefano.mancin@humanitas.it

**Keywords:** body mass index, obesity, healthy lifestyle, sociodemographic factors, survey, Spain

## Abstract

Aim: This cross-sectional study examines sociodemographic, nutritional, and lifestyle factors affecting Body Mass Index (BMI) in the Spanish population, with a particular emphasis on obesity. Methods: A sample of 22,181 Spanish residents aged 18 years and older was recruited through digital and physical channels from August 2020 to November 2021. Data were collected using the validated NutSo-HH questionnaire, which includes sections on sociodemographic information, health perceptions, eating habits, and lifestyle factors. Results: Among respondents, 661 (3%) were underweight (BMI < 18.5 kg/m^2^), 14,562 (65.7%) were normal weight (18.5 kg/m^2^ ≤ BMI ≤ 25 kg/m^2^), 4825 respondents (21.8%) were overweight (25 kg/m^2^ < BMI ≤ 30 kg/m^2^), and 2133 (9.6%) were obese (BMI > 30 kg/m^2^), with significant differences across these groups in relation to diet and lifestyle behaviors. Principal Component Analysis (PCA) was employed to identify the primary variables influencing obesity, revealing that poor dietary habits (frequent consumption of fast food, fried foods, and ultra-processed items) were negatively correlated with healthy behaviors such as regular fish consumption and physical activity. The PCA plot indicated notable distinctions based on educational attainment and age, with individuals with lower educational levels displaying poorer nutritional patterns and younger participants exhibiting higher fast food consumption and poorer sleep quality. Statistical analyses confirmed that sociodemographic factors, including age, education, and income level, significantly influenced BMI. Some differences were also found according to the place of residence. Conclusions: These findings underscore the need for targeted interventions that address both sociodemographic and lifestyle factors to mitigate obesity risk in Spain.

## 1. Introduction

Obesity represents a growing global health crisis with significant implications for public health and healthcare systems [1]. The World Health Organization (WHO) estimates that over 2.5 billion adults worldwide are overweight, with more than 890 million classified as obese [2], a condition closely linked to chronic diseases such as type 2 diabetes, cardiovascular conditions, and certain cancers [3]. In Europe, nearly 60% of adults are affected by overweight and obesity, with the prevalence steadily increasing across many countries due to notable shifts in dietary habits and lifestyle patterns [4].

Obesity is a complex, multifactorial phenomenon in which sociodemographic and environmental variables intersect with dietary choices and daily habits, creating a diverse and challenging landscape [5]. In Spain, the obesity epidemic reflects global trends but also reveals significant regional disparities, particularly in rural areas. Although Spain has traditionally followed the Mediterranean diet, known for its protective health benefits, recent decades have seen a decline in its adoption, reflecting a broader global shift toward diets high in ultra-processed foods, sugars, and fats [6]. Rural areas are particularly affected, with higher obesity rates linked to limited access to healthy foods, healthcare services, and physical activity opportunities. These regional disparities are compounded by cultural habits that promote less physical activity and reliance on high-calorie traditional meals [7]. While rural populations in other countries also face similar challenges, the unique socioeconomic and cultural context of Spain further exacerbates these issues, highlighting the need for regionally tailored public health interventions. This dietary transition, combined with socioeconomic disparities, emphasizes the urgent need for tailored nutritional interventions that address both regional and socioeconomic factors [8]. Additionally, sedentary lifestyles, reduced physical activity, and disrupted sleep-wake cycles further exacerbate this risk [9]. A cornerstone of obesity prevention lies in diet, which influences not only the energy balance but also the key metabolic and inflammatory processes underpinning fat accumulation [10]. Daily dietary choices determine the overall caloric intake and nutritional quality of consumed foods, which is crucial for maintaining a healthy body weight [11]. Diets rich in fruits, vegetables, whole grains, lean proteins, and unsaturated fats promote balanced energy intake while providing essential nutrients like vitamins, minerals, antioxidants, and dietary fiber. These nutrients enhance metabolic regulation and reduce chronic inflammation associated with obesity [12]. Conversely, excessive consumption of added sugars, trans fats, and salt, characteristic of contemporary Western diets, is strongly linked to an increased obesity risk [8]. Moreover, Spain’s dietary transition away from the Mediterranean model toward a more Westernized diet warrants attention, as it directly impacts public health and obesity prevalence. This shift also coincides with growing socioeconomic inequalities that influence food access and quality across different population groups. Ultra-processed foods, sugar-sweetened beverages, and foods high in saturated fats contribute to caloric excess and disrupt hunger and satiety signals, often leading to overeating [13]. These nutrient-poor foods can create a state of relative malnutrition despite high caloric intake, known as “over-nutrition”, which exacerbates visceral fat accumulation—a key predictor of metabolic complications such as insulin resistance and dyslipidemia [14,15]. Beyond food quality, eating behaviors play a critical role in weight regulation. Regular meal patterns, such as consuming breakfast and main meals at consistent times, are associated with better weight management. Breakfast, in particular, helps modulate daily metabolism, prevent glycemic spikes, and reduce high-calorie snacking [16]. In contrast, habits like skipping meals, late-night eating, or consuming large meals before bedtime disrupt energy metabolism and contribute to weight gain [17]. Mindful eating, which involves paying attention to the act of eating and recognizing hunger and satiety cues, is another key factor in obesity prevention. Interventions promoting mindful eating have proven effective in improving portion control, reducing overeating, and fostering a healthier relationship with food [18]. Psychological factors, such as stress and emotional regulation, also significantly influence dietary choices, often leading to dysfunctional behaviors like emotional eating, which contribute to weight gain [19]. Given the complexity of obesity, advanced analytical tools like principal component analysis (PCA) are invaluable for synthesizing and interpreting the multitude of contributing factors [20]. PCA identifies key dietary, behavioral, and environmental variables influencing body weight, offering a more integrated understanding of obesity dynamics. For instance, PCA can reveal how specific dietary patterns, such as frequent consumption of fried or ultra-processed foods, interact with behavioral factors like sleep quality and physical activity levels to determine obesity risk [21]. This study examines the sociodemographic, nutritional, and lifestyle factors influencing BMI in Spain. Specifically, it seeks to identify the key determinants of obesity through a cross-sectional analysis of diverse variables, such as dietary habits, physical activity, sleep patterns, and socioeconomic characteristics. These findings will provide a comprehensive understanding of obesity dynamics in Spain, emphasizing the need for tailored public health interventions. The justification for this research lies in the urgent need to address the increasing prevalence of obesity in Spain, particularly in light of its economic and social consequences. Obesity not only increases the burden on healthcare systems but also reduces the quality of life and productivity. By focusing on Spain, a country with unique dietary and cultural traditions undergoing significant change, this study offers valuable insights that can inform public health policies and interventions at both national and regional levels.

## 2. Materials and Methods

### 2.1. Study Design and Criteria

A cross-sectional study was carried out on the Spanish population residing in Spain. Subjects over 18 years of age were included, while those with chronic diseases that could affect their eating habits and those who were experiencing situations that temporarily altered their usual diet, such as hospitalization or imprisonment, were excluded.

### 2.2. Ethical Considerations

The ethical guidelines of the Declaration of Helsinki [22] were followed for the study, which was approved by the Research Ethics Committee of the Catholic University of Valencia (approval code UCV/2019-2020/152, 18 June 2020).

Before the questionnaire was administered, all participants were provided with information about the purpose of the study, as well as anonymous data collection and analysis. Respondents expressed their informed consent to participate in the study to authorize data handling and publication of the results.

### 2.3. Instrument

The validated questionnaire NutSo-HH (Nutritional and Social Healthy Habits Scale) was used for data collection [23]. NutSo-HH scale was developed and validated in two phases; during the first phase, conceptualization, design, cognitive and expert review, and pilot testing were carried out. Specifically, a panel of experts (N = 7), including a nutritionist, two family doctors, two psychologists, a social educator, and a communication expert, assessed content validity. Face validity was assessed through a pilot study involving 53 individuals with characteristics similar to the study population. In the second phase, the scale was applied to a sample of 571 Spanish young adults using non-probability sampling on social networks. The NutSo-HH consists of 23 items that assess eating habits, physical activity, sleep, and alcohol consumption. Confirmatory factor analysis, validity analysis, comparison with other instruments, and reliability analysis were performed, and the scale showed adequate psychometric properties. The omega coefficients ranged from 0.521 to 0.815.

This questionnaire includes several sections; the first one collects sociodemographic data of the respondents and health data such as weight, height, or self-perceived level of health. The second part focuses on analyzing habits and trends related to the respondents’ body perception and possible symptoms of eating disorders. The main and most extensive part focuses on the consumption frequencies of several food groups. The fourth part collects information on social habits such as alcohol consumption, smoking, or going out at night, while the last part collects data on sports habits.

### 2.4. Data Collection

Potential participants were contacted by email or social networks, especially through the Instagram account @elretonutricional, which was specifically created for this research. The Instagram account was used to contact several healthcare professionals and influencers who, after learning about the project, supported the dissemination of the questionnaire through their channels and networks. The researchers also utilized their networks on LinkedIn, Twitter, WhatsApp, and Facebook to circulate the questionnaires to potential respondents. Additionally, the survey was physically disseminated with the support of various public establishments, such as pharmacies and tobacconists.

Participants were encouraged to share the link to the online survey with their contacts using the ‘snowball’ method [24], which involves relying on participants who meet the study criteria to identify others who also meet the criteria. This process was repeated until the desired sample size was reached. The study conformed to the STROBE guidelines [25]. The data collection period was from August 2020 to November 2021.

### 2.5. Variables

The questionnaire encompasses a broad range of sociodemographic factors, collecting data on sex, age, birthplace, current residence, employment details, education level, income bracket, and living arrangements (e.g., living alone, with others, or in the family home). It also includes anthropometric and health-related information, such as self-reported weight and height, and self-assessed health status.

For data analysis, sociodemographic variables were divided into groups using the following criteria:

- Sex: divided between men and women
- Age: divided between youth (≤30 years old) and adults (>30 years old).
- Level of education: divided between basic education (which includes subjects with no education, those with only primary or compulsory secondary education, and those with baccalaureate or vocational education) and higher education (which includes respondents with a university degree, master’s degree, or PhD).
- Income level: divided between Low Income (those subjects with a gross monthly income of less than 2200€ per household) and medium-high income (income of more than 2200€ gross/monthly).
- Municipality size: Categorized by population size into three groups: small municipalities (fewer than 2000 inhabitants), medium-sized towns (2000 to 10,000 inhabitants), and cities (more than 10,000 inhabitants).
- Living arrangements: Classified as living alone or not living alone.
- Family life: Distinguished between living with family and living without family.
- Spanish communities: Grouped according to different regions of Spain.

To evaluate diet-related health conditions, symptoms of eating disorders, and nutritional habits, the survey employed the 23-item Nutritional and Social Healthy Habits (NutSo-HH) scale. This instrument assesses the frequency of food consumption, sedentary activities, physical exercise, and health-related social behaviors, including sleep patterns, smoking, and alcohol use.

To analyze habit variables, a categorization approach was applied, as described in previous studies [26,27], converting qualitative variables into nominal quantitative ones. For each variable, a Likert scale score from 1 to 4 was assigned, where 1 corresponded to the lowest frequency of consuming food or performing a lifestyle habit, while a score of 4 corresponded to the highest or most frequent value.

Most food consumption habits were incorporated into the calculation of the Healthy Nutrition Index for the Spanish population (IASE) using a simplified version of the scale validated by Norte and Navarro [28]. This version categorizes items such as “fruit”, “vegetables”, “meat”, “dairy”, “cereals”, “pulses”, and “soft drinks”. This index evaluates the frequency of recommended daily and weekly food intake and the diversity of dietary choices, an essential component of a balanced diet. Scores are assigned based on adherence to dietary guidelines from the Spanish Society of Community Nutrition (SENC) [29], with a maximum possible score of 73. Nutritional habits are classified as follows:

Healthy: Scores between 58.4 and 73
Needs changes: Scores between 36.5 and 58.4
Unhealthy: Scores below 36.5

Additionally, the BMI groups were analyzed as follows:

Underweight: BMI < 18.5 kg/m^2^
Normal weight: 18.5 kg/m^2^ ≤ BMI ≤ 25 kg/m^2^
Overweight: 25 kg/m^2^ < BMI ≤ 30 kg/m^2^
Obesity: BMI > 30 kg/m^2^

### 2.6. Data Analysis

Initially, the dataset was refined by removing erroneous or atypical entries, including inaccurate responses and outlier data for height and weight. Specifically, BMI values below 14 and above 40 were filtered out. After cleaning the data, a normality assessment was conducted using the Shapiro-Wilk test, which confirmed that none of the variables followed a normal distribution. This finding was further supported by Q-Q plots [30]. Consequently, the Chi-Square Test was employed for categorical variables, while the non-parametric Mann−Whitney U Test or Kruskal−Wallis test was used for ordinal or numerical variables in the independent samples. To analyze differences between multiple groups, the Dwass-Steel-Critchlow-Fligner pairwise comparison test was applied [31]. Sociodemographic categorical variables are presented as counts and percentages, while continuous variables are reported as means and standard deviations. A significance level of 0.05 was set for all statistical tests.

Principal Component Analysis (PCA) was applied as an unsupervised machine learning approach to reduce the dimensionality of the dataset. This method consolidates a large set of variables into fewer principal components that capture most of the variance in the original data. Principal components were calculated as linear combinations of the initial variables. All statistical analyses were conducted using Jamovi Version 2.3.28.0 [32]. PCA graphs were generated in Jamovi, while other charts were created using Microsoft Excel.

## 3. Results

### 3.1. Sociodemographic Characteristics

The sample included 80.8% women, who were on average 34.9 years old, and 68.3% with a bachelor’s degree or higher. The distribution by income level was even: 43.9% of respondents had a low-income level versus 47.9% who had a medium-high income, and 8.3% preferred not to answer the question. Table 1 describes the sociodemographic characteristics of the sample.

### 3.2. BMI Analysis Divided into 4 Groups

**Figure 1:**
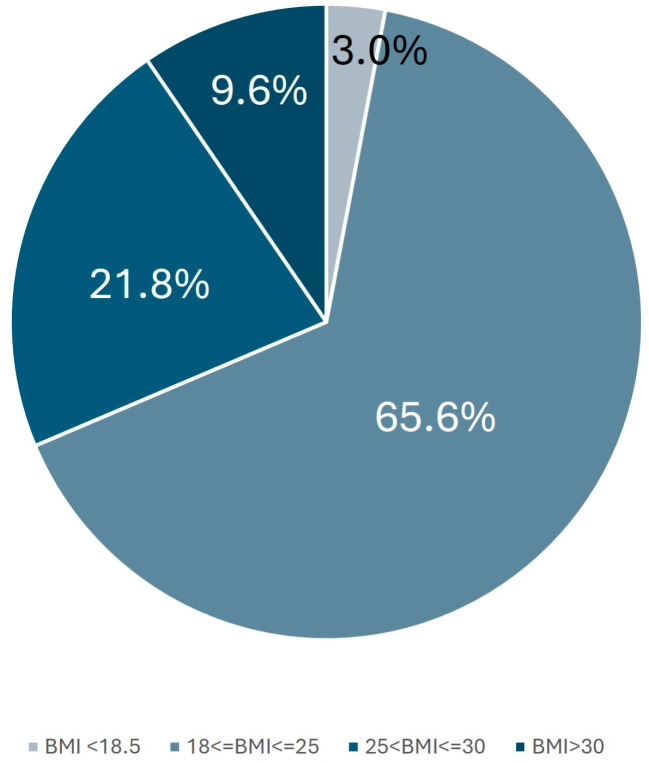
Distribution of the population according to BMI.

Analyzing the BMI distribution within the population (Figure 1), 661 individuals (3%) were classified as underweight (BMI < 18.5 kg/m^2^). The majority, 14,562 individuals (65.6%), fell within the normal weight range (18.5 kg/m^2^ ≤ BMI ≤ 25 kg/m^2^). Meanwhile, 4825 respondents (21.8%) were categorized as overweight (25 kg/m^2^ < BMI ≤ 30 kg/m^2^), and 2133 individuals (9.6%) were classified as obese (BMI > 30 kg/m^2^).

Analyzing the variables related to nutritional, social, and lifestyle habits across the four BMI groups (underweight, normal weight, overweight, and obesity) reveals statistically significant differences among nearly all groups for most variables (Table 2). The only exception was juice consumption, which did not vary according to BMI. In general, the healthiest scores are observed in the normal-weight group, with a slight decline among those in the overweight category. However, in the group with obesity, scores for all variables worsened significantly and more markedly compared to the other groups.

Table 3 shows the absolute value and percentage of people belonging to each of the BMI groups with respect to the different sociodemographic variables. There are significantly more underweight and normal-weight women than men, while there are more overweight men and no differences between the sexes in the Obesity group.

With respect to age, there are more underweight and normal-weight young people and more adults with overweight and obesity. For the level of education, no significant differences are found in the underweight category; most of the people with normal weight have higher education, while overweight and obesity are more prevalent among people with basic education. For income level, the differences are smaller but significant for all groups, showing that people with low income have an unbalanced BMI; there are more people with low weight and obesity.

With respect to the size of the municipality of residence, obesity appears to be more prevalent in small towns than in cities. Among people living in families, there is a higher prevalence of overweight and obesity. Finally, no statistically significant differences were observed with respect to living alone or being accompanied by any group.

**Figure 2:**
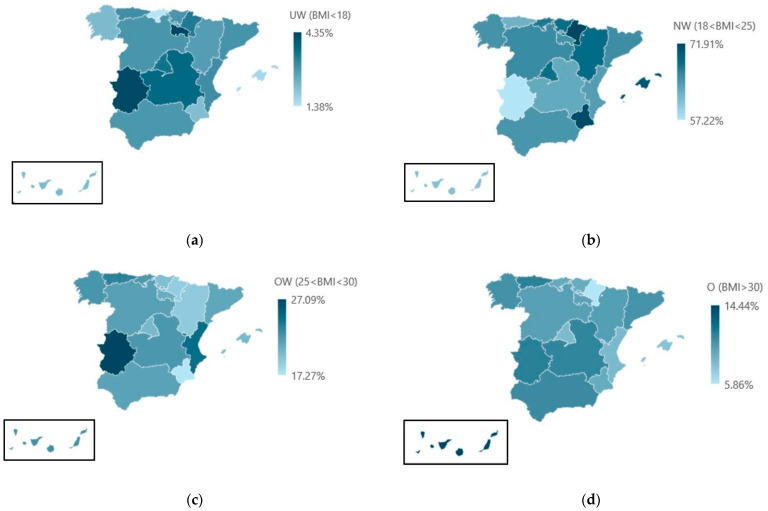
Analysis of the frequency of occurrence of a BMI category in different regions of Spain. (**a**) Under Weight (UW), (**b**) Normal Weight (NW), (**c**) Over Weight (OW), and (**d**) Obesity (O).

**Figure A1:**
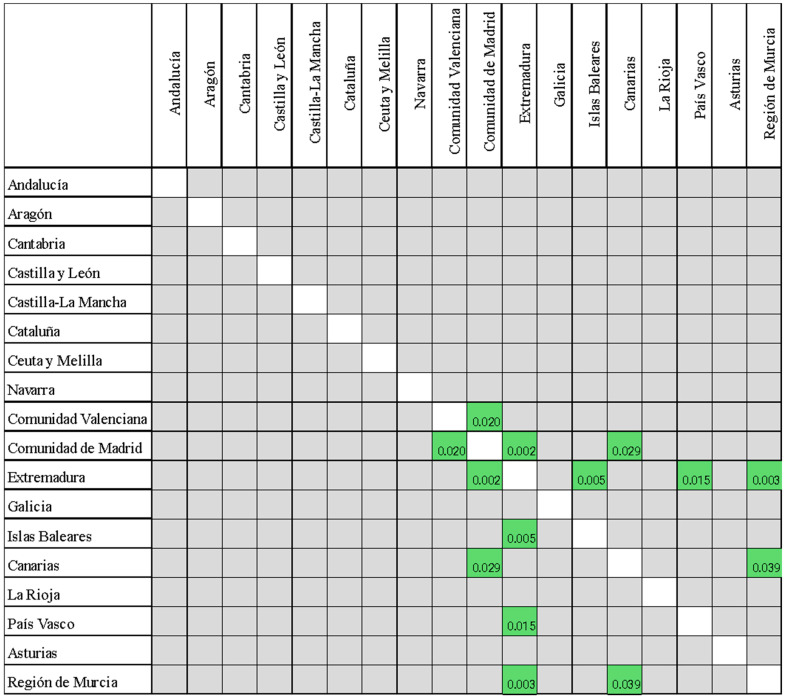
*p*-values of the frequency of normal-weight people for regions of Spain comparison using the Dwass-Steel-Critchlow-Fligner pairwise test. (NOTE: In the gray boxes, the *p*-value is not statistically significant >0.05. Green cells indicate values with statistically significant differences (*p* < 0.05). While the white cells corresponding to the diagonal represent the comparison of the variable with itself, which is meaningless).

**Figure A2:**
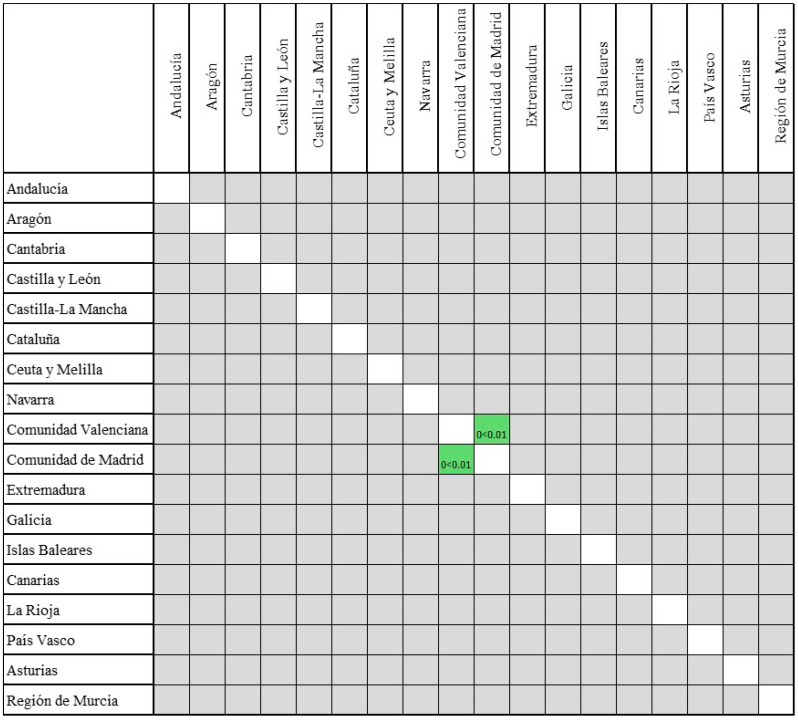
*p*-values of the frequency of overweight people for regions of Spain comparison using the Dwass-Steel-Critchlow-Fligner pairwise test. (NOTE: In the gray boxes, the *p*-value is not statistically significant >0.05. Green cells indicate values with statistically significant differences (*p* < 0.05). While the white cells corresponding to the diagonal represent the comparison of the variable with itself, which is meaningless).

**Figure A3:**
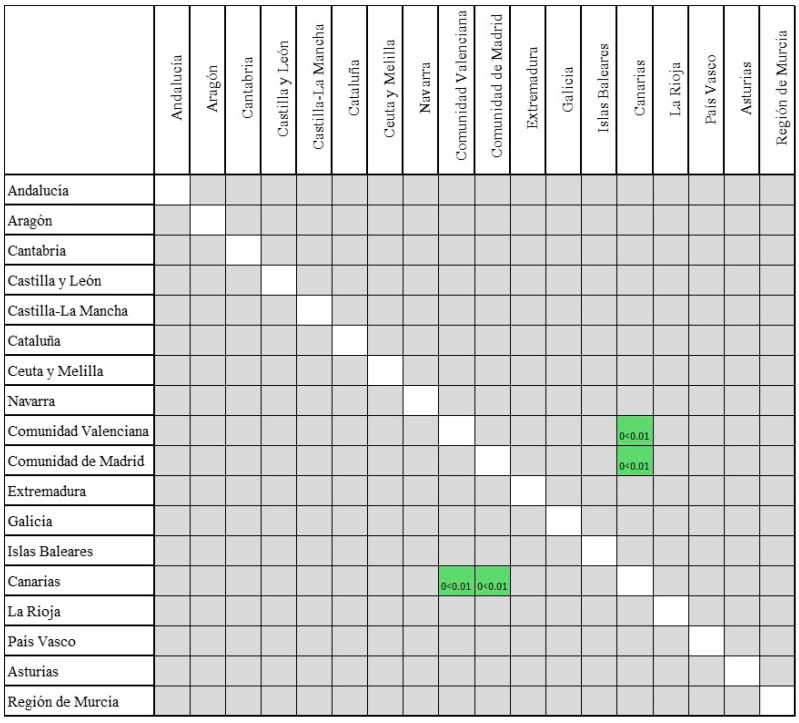
*p*-values of the frequency of people with obesity for regions of Spain comparison using the Dwass-Steel-Critchlow-Fligner pairwise test. (NOTE: In the gray boxes, the *p*-value is not statistically significant >0.05. Green cells indicate values with statistically significant differences (*p* < 0.05). While the white cells corresponding to the diagonal represent the comparison of the variable with itself, which is meaningless).

Table 4 and Figure 2a–d show the frequency of occurrence of a BMI category in different regions of Spain. They show in detail the number (N) and percentage (%) of people with BMI in each category. For the underweight group, no statistically significant differences were found with respect to the Spanish region of residence (*p* = 0.335), while for the groups NW, OW, and O, there are regions where the incidence of this category is different from the rest (*p* < 0.001). Figure A1, Figure A2 and Figure A3 in Appendix A report the *p*-values for each pair of regions, allowing us to analyze in detail the geographical areas in which these differences are found.

### 3.3. Analysis of the Respondents with Obesity

The sample of the population with obesity had the sociodemographic characteristics described in Table 5. The percentage of men and women is very similar to that of the whole population (Table 1); the mean age of men with obesity is higher than that of women (42.0 vs. 37.7), and obesity is more prevalent among people with low income (53.2%) than among those with medium-high income (46.8%).

Focusing on people with obesity (N = 2133), a principal component analysis (PCA) was performed to reduce the dimensionality of the variables and identify the most influential factors; its key findings will be outlined.

The selection of the appropriate number of principal components can be seen in Appendix B.

**Figure 3:**
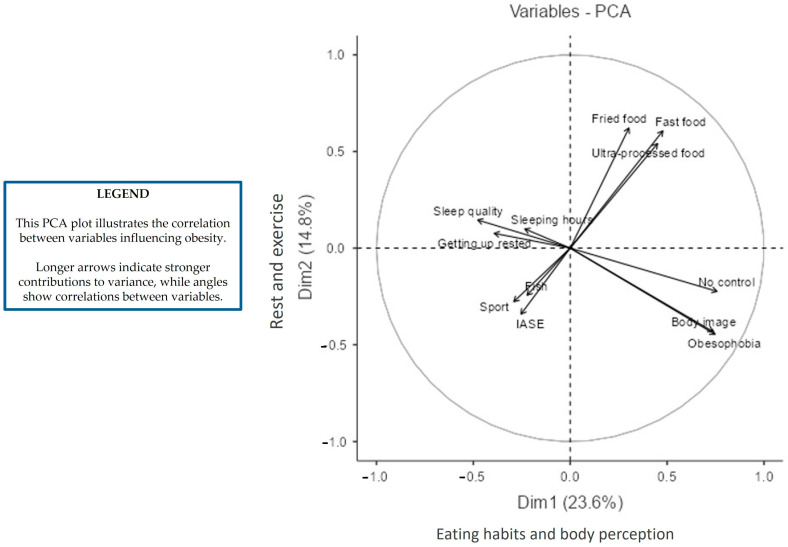
Variables plot of the PCA model. (NOTE: Only the top twelve variables contributing the most to the PCA model are represented).

Figure 3 shows a plot of the variables in the PCA model. In this graph, variables are grouped according to their relationship with the dimensions. Dimension 1 is more related to eating habits and body perception, while Dimension 2 is more linked to rest and exercise.

The following variables: no control over food intake, obesophobia, and body image are highly correlated (the arrows are at 0°) and contribute the most to the model (they are the longest). In contrast, no correlation can be observed (because the arrows are at 90°) between these variables and fried food, fast food, and ultra-processed dishes, which are the other three variables that contribute the most to explain the variability of the data.

The following variables: no control over food intake, obesophobia, and body image are negatively correlated with sleep quality, sleeping hours, and waking up rested (arrows are at 180°). In other words, respondents with obesophobia, no control over food intake, or body image concerns sleep less and are worse than those without these feelings.

Finally, unhealthy food variables correlate negatively with IASE and fish consumption, as is logical, but also with the time devoted to physical activity, indicating how food is strictly related to people’s sporting activity.

If we study in detail the twelve variables of nutrition and lifestyle habits that most influence obesity with respect to the sociodemographic variables, we find the behavior described in Table 6.

We observe that with respect to sex, there are statistically significant differences for almost all the variables and where women seem to eat better (higher consumption of fish and lower consumption of fried and ultra-processed foods) than men, but in contrast, have greater concern about gaining weight, body image, or not having control over the food consumed.

With respect to the age variable, adults have a higher IASE score, lower consumption of fried food, fast food, and ultra-processed food, and higher consumption of fish, but seem to sleep fewer hours and with poorer quality compared to young people.

Analyzing the education level, people with higher education have better nutrition (a significantly higher IASE score, consume less fried and ultra-processed food, and more fish), sleep better, and wake up more rested.

Finally, for the variables of income level and living with or without a family and living alone or accompanied, statistically significant differences are found in a few variables, not allowing for the definition of a clear pattern of variable behavior.

**Figure 4:**
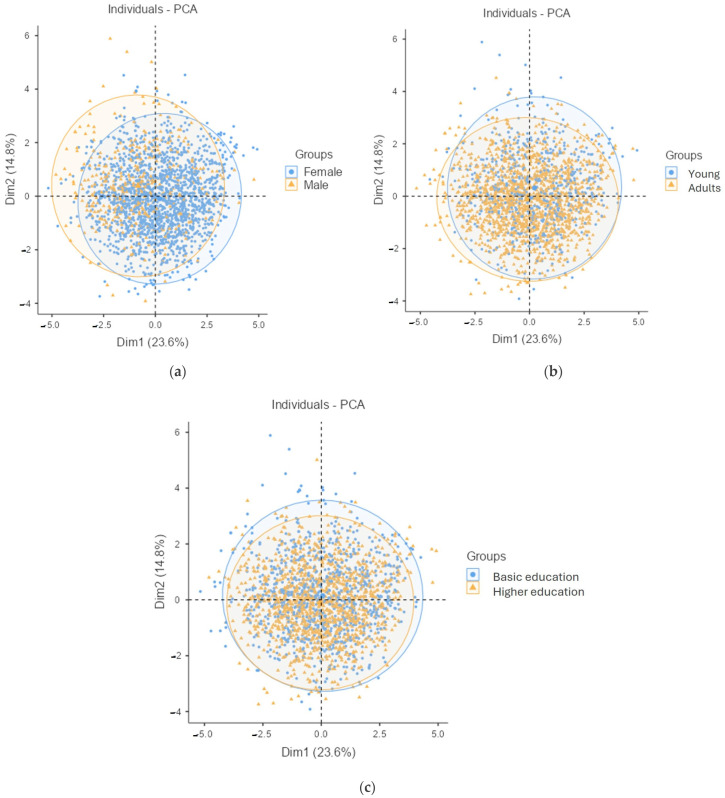
Individuals plot of the PCA model depending on the sociodemographic variables. (**a**) Sex; (**b**) Age; (**c**) Educational level.

When individuals with obesity are represented in the two dimensions of the PCA and color-coded according to the sociodemographic variables that most distinctly separate groups (sex, age, and education level), the graphs in Figure 4a–c are obtained.

Figure 4a illustrates the distribution of individuals grouped by sex (yellow dots: men; blue dots: women). The blue dots, representing women, are predominantly shifted toward the lower-right quadrant, where the variables obesophobia, no control over food intake, and body image are located. This indicates that women with obesity score higher on these variables, reflecting greater attention to these aspects. Conversely, the yellow dots for men are concentrated in the upper-left quadrant, where the variables related to rest are located, suggesting that men generally experience better rest than women. These findings align with the behaviors detailed in Table 6.

Figure 4b shows the distribution of individuals with obesity categorized by age (blue dots: young people; yellow dots: adults). The blue dots, representing younger individuals, cluster in the upper quadrants, where variables related to unhealthy food consumption and poor rest habits are located. In contrast, the yellow dots, representing adults, shift to the lower-left quadrant, where variables such as IASE (Index of Adherence to a Spanish Healthy Diet) and fish consumption are located. This suggests that adults have better nutritional habits than younger individuals but experience fewer hours and lower quality of sleep.

Figure 4c displays individuals with obesity based on their educational level (blue dots: basic education; yellow dots: higher education). The blue dots, representing individuals with a basic education, are predominantly located in the upper-right quadrant, which includes variables associated with unhealthy food consumption. This confirms that individuals with lower educational attainment generally exhibit poorer nutritional habits compared to those with higher education.

## 4. Discussion

The findings of this study align with existing evidence on the influence of sociodemographic, nutritional, and lifestyle factors on BMI and obesity rates across diverse populations. The higher prevalence of obesity among individuals with lower education and income reflects widespread social disparities, as documented in studies from Europe, the U.S., and other Mediterranean countries [33]. In Spain, these disparities are particularly pronounced in rural areas, where limited financial resources and access to healthy food exacerbate obesity rates. This trend is mirrored in other Mediterranean countries, such as Greece, which have also seen a shift away from traditional Mediterranean diets in favor of ultra-processed foods, contributing to rising obesity rates [34]. However, Spain’s socioeconomic challenges, including higher unemployment rates in certain regions, make these disparities even more acute compared to countries like Italy, where similar shifts in dietary patterns are occurring. Despite efforts to promote local food systems and community-based initiatives, Italy is also grappling with the rising prevalence of obesity as a result of these dietary transitions [35,36].

As in the U.S., limited financial resources in Spain often restrict access to nutritious foods, leading to a greater reliance on inexpensive, nutrient-poor, ultra-processed options. For example, U.S.-based research has demonstrated that restricted access to fresh fruits and vegetables in economically disadvantaged areas is directly linked to a higher obesity risk, accompanied by an increased intake of processed foods and sugary beverages [37,38]. In Spain, similar patterns are observed, with lower-income individuals being more likely to consume fast foods and ultra-processed products, which are often marketed more aggressively in lower-income areas [6,7].

The observation that obesity rates are higher in smaller Spanish towns compared to urban areas is consistent with European studies indicating that rural settings may have fewer opportunities for structured physical activity and limited access to public recreational spaces [39]. Additionally, rural areas often face barriers, such as reduced access to healthcare services and nutrition education, further impacting dietary habits and physical activity levels [40].

It is important to consider the differences in obesity prevalence between rural and urban areas. Previous studies have highlighted that rural areas tend to have higher obesity rates compared to urban areas, attributed to factors such as reduced access to physical activity facilities, limited availability of healthy foods, and socioeconomic disparities [23]. However, in Spain, some research suggests that rural-urban disparities in obesity may be less pronounced, underscoring the need for further studies to fully understand these dynamics [24]. Moreover, cultural and lifestyle differences between rural and urban areas can influence dietary habits and physical activity levels, contributing to variations in obesity rates. For instance, urban areas may offer greater access to unhealthy food options and limited opportunities for physical activity due to fast-paced lifestyles and built environments that discourage movement [25]. Conversely, rural areas may face barriers related to access to fresh and healthy foods, as well as limited infrastructure for physical activity [26]. These differences underscore the importance of developing targeted public health interventions that account for local specificities, promoting healthy eating habits and physical activity in both rural and urban settings.

Behaviorally, this study highlights a strong association between frequent consumption of fried and ultra-processed foods and higher BMI, a link extensively supported by the existing literature. Diets high in ultra-processed foods, sugars, and saturated fats contribute to energy imbalances that promote weight gain and increase obesity risk [41]. Moreover, individuals with obesity who reported obesophobia, lack of control over food intake, and body image concerns were found to have poorer sleep quality. This is consistent with international research showing that poor sleep quality exacerbates stress and increases the likelihood of disordered eating behaviors [42]. Psychological stress linked to body dissatisfaction further impairs food intake control, compounding challenges in weight regulation [43].

Nutritional and sleep-related variables play a significant role in determining BMI. PCA explains approximately 38% of the variance, with key factors including obesophobia, sleep quality, and ultra-processed food consumption. This aligns with prior research using PCA to identify the behavioral determinants of obesity, reinforcing the importance of sleep and emotional regulation in weight management [44]. Public health initiatives that incorporate strategies for improving emotional regulation and sleep quality could provide effective interventions for reducing obesity rates, as supported by international findings [45].

This study also highlights the urgent need for public health measures to address inequities in access to nutritious foods and safe spaces for physical activity, particularly in vulnerable populations. Strategies such as subsidizing fresh produce, enhancing food distribution networks, and implementing culturally tailored nutrition education programs could reduce dependency on ultra-processed products in underserved communities [33]. Similarly, expanding safe and accessible recreational spaces in both rural and urban areas could foster physical activity and improve overall health outcomes [46].

In line with WHO recommendations, targeted efforts to limit the availability of ultra-processed foods in disadvantaged communities and promote mindful eating practices are essential [47]. Addressing the interconnected dimensions of diet, lifestyle, and psychosocial factors through comprehensive public health initiatives can significantly reduce obesity rates. For instance, community-based programs could be developed in rural and underserved areas, offering education on nutrition and healthy eating, while also promoting physical activity. Subsidies for healthy food could be introduced to make nutritious options more affordable in these regions, along with the establishment of farmer markets and local food cooperatives to increase access to fresh produce. Additionally, education campaigns focusing on obesophobia and sleep hygiene are crucial for tackling the psychological and behavioral aspects of obesity. Integrating interventions to enhance sleep quality and manage stress into these strategies presents additional opportunities to tackle obesity’s multifactorial nature, paving the way for sustainable solutions to this critical public health challenge.

Future research should incorporate longitudinal data to better establish causality and further investigate psychological factors, such as stress and social support, which play a crucial role in influencing dietary and lifestyle behaviors.

### Strength and Limitations

The strengths of this study include its large sample size and representativeness of the Spanish population, which allows for a detailed and generalizable analysis of the sociodemographic, nutritional, and lifestyle factors associated with BMI and obesity. The application of PCA effectively reduced data complexity, emphasizing the most influential variables and simplifying the interpretation of the results. Moreover, the inclusion of diverse sociodemographic variables provided a more nuanced perspective on the behaviors and conditions linked to obesity, offering valuable insights for designing targeted public health interventions.

However, this study has notable limitations. The cross-sectional design prevents the establishment of causal relationships between the factors analyzed and obesity, underscoring the need for longitudinal research to validate the observed associations, as well as the potential use of predictive modeling to assess the long-term impact of interventions on obesity trends across different socioeconomic groups. The reliance on self-reported data for dietary and lifestyle variables introduces potential response and recall biases, which can affect the accuracy of the findings. Additionally, social desirability bias may have also influenced the reported data. Finally, while the study examined a broad range of factors, it did not account for key psychological variables, such as stress and social support, which are known to significantly impact eating behaviors and weight management. These factors warrant further investigation in future research.

## 5. Conclusions

This study highlights the multifaceted influence of sociodemographic, nutritional, and lifestyle factors on BMI and obesity in the Spanish population. These findings highlight the critical role of poor dietary habits and disparities in obesity prevalence in relation to education level, income level, and geographic context. A comparison between rural and urban populations revealed nuanced differences in the determinants of obesity, with rural areas facing challenges such as limited access to healthy foods and physical activity infrastructure, while urban areas struggle with environments that promote sedentary behaviors and increased consumption of high-calorie foods. Leveraging PCA, this study enabled the identification of key determinants of obesity, such as the impact of poor sleep quality and stress-related eating behaviors, which were particularly pronounced in women and individuals from disadvantaged socioeconomic backgrounds. To address the growing prevalence of obesity in Spain, it is essential to implement culturally and regionally tailored public health policies focusing on increasing access to affordable and nutritious foods, improving opportunities for physical activity in both rural and urban areas, and addressing psychological factors such as stress and body image issues. This study emphasizes the need for targeted public health policies that focus on dietary education and physical activity infrastructure, particularly in rural and disadvantaged communities. Future efforts should also address psychological factors, such as stress and body image, to provide holistic interventions.

## Figures and Tables

**Table 1:** Sample sociodemographic characteristics (N = 22,181).

		Mean (SD) or N (%)
Sex	Male	4251 (19.2%)
	Female	17,930 (80.8%)
Age (years)	Age total	34.9 (11.7)
	Age male	36.5 (13.4)
	Age female	34.5 (11.2)
	Young (≤30 years)	9692 (43.7%)
	Adults (>30 years)	12,489 (56.3%)
Education level	Basic Education	7027 (31.7%)
	Higher Education	15,154 (68.3%)
Income Level	Low	9727 (43.9%)
	Medium-high	10,616 (47.9%)
	No answer	1839 (8.3%)
Municipality	<2000	1014 (4.6%)
	2000–10,000	3587 (16.2%)
	>10,000	17,580 (79.3%)
Living arrangement	Living alone	2202 (9.9%)
	Not living alone	19,979 (90.1%)
Family life	Living with family	16,732 (75.4%)
	Living without family	5449 (24.6%)
Spanish communities	Andalusia	2617 (11.8%)
	Aragon	652 (2.9%)
	Asturias	661 (3.0%)
	Balearic Islands	488 (2.2%)
	Basque Country	925 (4.2%)
	Canary Islands	637 (2.9%)
	Cantabria	217 (1.0%)
	Castilla-La Mancha	729 (3.3%)
	Castile and León	1276 (5.8%)
	Cataluña	3540 (16.0%)
	Ceuta and Melilla	28 (0.1%)
	Community of Madrid	3882 (17.5%)
	Extremadura	395 (1.8%)
	Galicia	1404 (6.3%)
	La Rioja	115 (0.5%)
	Navarre	324 (1.5%)
	Region of Murcia	417 (1.9%)
	Valencian Community	3874 (17.5%)

**Table 2:** Nutritional, social, and lifestyle variables for the four different BMI groups.

Numerical Variables	Under Weight (UW) BMI < 18.5 kg/m^2^	Normal Weight (NW) 18.5 kg/m^2^ < BMI < 25 kg/m^2^	Over Weight (OW) 25 kg/m^2^ < BMI < 30 kg/m^2^	Obesity (O) BMI > 30 kg/m^2^	*p*-Value ^$^
	Mean (SD)	Mean (SD)	Mean (SD)	Mean (SD)	
IASE (Healthy Nutrition Index for the Spanish population)	52.6 (10.4)	54.3 (9.69)	53.3 (9.77)	51.4 (10.6)	UW-NW (*p* < 0.001)
					UW-OW (*p* = 0.625)
					UW-O (*p* = 0.0.38)
					NW-OW (*p* < 0.001)
					NW-O (*p* < 0.001)
					OW-O (*p* < 0.001)
Fried food	2.33 (0.91)	2.21 (0.81)	2.29 (0.81)	2.37 (0.84)	UW-NW (*p* = 0.020)
					UW-OW (*p* = 0.963)
					UW-O (*p* = 0.390)
					NW-OW (*p* < 0.001)
					NW-O (*p* < 0.001)
					OW-O (*p* = 0.001)
Fast food	2.32 (0.86)	2.33 (0.76)	2.43 (0.76)	2.55 (0.79)	UW-NW (*p* = 0.779)
					UW-OW (*p* = 0.002)
					UW-O (*p* < 0.001)
					NW-OW (*p* < 0.001)
					NW-O (*p* < 0.001)
					OW-O (*p* < 0.001)
Ultra-processed food	2.37 (1.01)	2.27 (0.94)	2.36 (0.94)	2.55 (1.01)	UW-NW (*p* = 0.051)
					UW-OW (*p* = 0.991)
					UW-O (*p* < 0.001)
					NW-OW (*p* < 0.001)
					NW-O (*p* < 0.001)
					OW-O (*p* < 0.001)
Fish	1.76 (0.53)	1.83 (0.50)	1.85 (0.50)	1.82 (0.53)	UW-NW (*p* = 0.200)
					UW-OW (*p* < 0.001)
					UW-O (*p* = 0.035)
					NW-OW (*p* = 0.019)
					NW-O (*p* = 0.871)
					OW-O (*p* = 0.065)
Water	3.26 (0.67)	3.40 (0.63)	3.42 (0.65)	3.47 (0.64)	UW-NW (*p* < 0.001)
					UW-OW (*p* < 0.001)
					UW-O (*p* < 0.001)
					NW-OW (*p* = 0.007)
					NW-O (*p* < 0.001)
					OW-O (*p* = 0.030)
Sugary soft drinks	1.36 (0.68)	1.33 (0.61)	1.47 (0.71)	1.62 (0.82)	UW-NW (*p* = 0.969)
					UW-OW (*p* < 0.001)
					UW-O (*p* < 0.001)
					NW-OW (*p* < 0.001)
					NW-O (*p* < 0.001)
					OW-O (*p* < 0.001)
Juice	1.26 (0.54)	1.25 (0.54)	1.23 (0.52)	1.22 (0.52)	*p* = 0.036
Coffee and energy drinks	1.53 (0.64)	1.70 (0.70)	1.79 (0.73)	1.72 (0.74)	UW-NW (*p* < 0.001)
					UW-OW (*p* < 0.001)
					UW-O (*p* < 0.001)
					NW-OW (*p* < 0.001)
					NW-O (*p* = 0.892)
					OW-O (*p* < 0.001)
Sedentary lifestyle	1.61 (0.82)	1.55 (0.81)	1.59 (0.85)	1.69 (0.93)	UW-NW (*p* = 0.200)
					UW-OW (*p* = 0.678)
					UW-O (*p* = 0.448)
					NW-OW (*p* = 0.272)
					NW-O (*p* < 0.001)
					OW-O (*p* < 0.001)
Self-perceived health	3.76 (0.92)	3.95 (0.78)	3.67 (0.84)	3.28 (0.91)	UW-NW (*p* < 0.001)
					UW-OW (*p* = 0.002)
					UW-O (*p* < 0.001)
					NW-OW (*p* < 0.001)
					NW-O (*p* < 0.001)
					OW-O (*p* < 0.001)
Sport	133 (179)	174 (181)	142 (173)	90.6 (136)	UW-NW (*p* < 0.001)
					UW-OW (*p* = 0.142)
					UW-O (*p* < 0.001)
					NW-OW (*p* < 0.001)
					NW-O (*p* < 0.001)
					OW-O (*p* < 0.001)
Sleeping hours	2.59 (0.75)	2.57 (0.71)	2.44 (0.74)	2.33 (0.80)	UW-NW (*p* = 0.857)
					UW-OW (*p* < 0.001)
					UW-O (*p* < 0.001)
					NW-OW (*p* < 0.001)
					NW-O (*p* < 0.001)
					OW-O (*p* < 0.001)
Getting up rested	2.55 (0.60)	2.56 (0.58)	2.52 (0.58)	2.43 (0.59)	UW-NW (*p* = 0.811)
					UW-OW (*p* = 0.806)
					UW-O (*p* < 0.001)
					NW-OW (*p* < 0.001)
					NW-O (*p* < 0.001)
					OW-O (*p* < 0.001)
Sleep quality	3.37 (1.10)	3.45 (0.99)	3.32 (1.03)	3.11 (1.09)	UW-NW (*p* = 0.481)
					UW-OW (*p* = 0.549)
					UW-O (*p* < 0.001)
					NW-OW (*p* < 0.001)
					NW-O (*p* < 0.001)
					OW-O (*p* < 0.001)
Smoking	1.24 (0.63)	1.21 (0.60)	1.27 (0.69)	1.30 (0.73)	UW-NW (*p* = 0.469)
					UW-OW (*p* = 0.894)
					UW-O (*p* = 0.504)
					NW-OW (*p* < 0.001)
					NW-O (*p* < 0.001)
					OW-O (*p* = 0.576)
Alcohol	1.59 (0.79)	1.75 (0.85)	1.85 (0.95)	1.64 (0.85)	UW-NW (*p* < 0.001)
					UW-OW (*p* < 0.001)
					UW-O (*p* = 0.734)
					NW-OW (*p* < 0.001)
					NW-O (*p* < 0.001)
					OW-O (*p* < 0.001)
Getting drunk	1.04 (0.21)	1.06 (0.28)	1.08 (0.34)	1.06 (0.30)	UW-NW (*p* = 0.625)
					UW-OW (*p* = 0.079)
					UW-O (*p* = 0.901)
					NW-OW (*p* = 0.002)
					NW-O (*p* = 0.857)
					OW-O (*p* = 0.025)
Night outings	1.23 (0.46)	1.20 (0.44)	1.17 (0.42)	1.13 (0.40)	UW-NW (*p* = 0.257)
					UW-OW (*p* < 0.001)
					UW-O (*p* < 0.001)
					NW-OW (*p* < 0.001)
					NW-O (*p* < 0.001)
					OW-O (*p* < 0.001)
Obesophobia	2.23 (1.47)	3.16 (1.36)	3.98 (1.32)	4.60 (1.24)	UW-NW (*p* < 0.001)
					UW-OW (*p* < 0.001)
					UW-O (*p* < 0.001)
					NW-OW (*p* < 0.001)
					NW-O (*p* < 0.001)
					OW-O (*p* < 0.001)
No control over food intake	2.03 (1.26)	2.55 (1.18)	3.14 (1.28)	3.72 (1.36)	UW-NW (*p* < 0.001)
					UW-OW (*p* < 0.001)
					UW-O (*p* < 0.001)
					NW-OW (*p* < 0.001)
					NW-O (*p* < 0.001)
					OW-O (*p* < 0.001)
Body image	3.22 (1.28)	3.44 (1.24)	3.85 (1.27)	4.37 (1.29)	UW-NW (*p* < 0.001)
					UW-OW (*p* < 0.001)
					UW-O (*p* < 0.001)
					NW-OW (*p* < 0.001)
					NW-O (*p* < 0.001)
					OW-O (*p* < 0.001)
Diagnosed Eating Disorder	0.08 (0.27)	0.03 (0.17)	0.03 (0.17)	0.08 (0.27)	UW-NW (*p* < 0.001)
					UW-OW (*p* < 0.001)
					UW-O (*p* = 0.996)
					NW-OW (*p* = 1.000)
					NW-O (*p* < 0.001)
					OW-O (*p* < 0.001)

^$^ Kruskal-Wallis Test; NOTE: Statistically significant relationships are shown in blue.

**Table 3:** Sociodemographic variables for the four different BMI groups.

Sociodemographic Variables	Under Weight (UW) BMI < 18.5 kg/m^2^		Normal Weight (NW) 18.5 kg/m^2^ < BMI < 25 kg/m^2^		Over Weight (OW) 25 kg/m^2^ < BMI < 30 kg/m^2^		Obesity (O) BMI > 30 kg/m^2^	
Men	21	0.49%	2403	56.53%	1403	33.00%	424	9.97%
Women	640	3.57%	12,159	67.81%	3422	19.09%	1709	9.53%
*p*-value	<0.001		<0.001		<0.001		0.379	
Young (≤30 years)	438	4.52%	7070	72.95%	1589	16.39%	595	6.14%
Adults (>30 years)	223	1.79%	7492	59.99%	3236	25.91%	1538	12.31%
*p*-value	<0.001		<0.001		<0.001		<0.001	
Low education	216	3.07%	4307	61.29%	1610	22.91%	894	12.72%
High education	445	2.94%	10,255	67.67%	3215	21.22%	1239	8.18%
*p*-value	0.576		<0.001		0.004		<0.001	
Low income	304	3.13%	6277	64.53%	2078	21.36%	1068	10.98%
Medium-high income	279	2.63%	6991	65.85%	2405	22.65%	941	8.86%
*p*-value	0.034		0.048		0.026		<0.001	
Small city	89	3.06%	2334	61.83%	790	22.58%	374	12.52%
Medium city	31	2.48%	627	65.07%	229	22.02%	127	10.43%
Big city	541	3.08%	11,601	65.99%	3804	21.64%	1632	9.28%
*p*-value	0.158		0.018		0.713		<0.001	
			Small city vs. Medium city (*p* = 0.139)				Small city vs. Medium city (*p* = 0.141)	
			Small city vs. Big city (*p* = 0.018)				Small city vs. Big city (*p* = 0.002)	
			Medium city vs. Big city (*p* = 0.539)				Medium city vs. Big city (*p* = 0.084)	
Living with family	471	2.81%	10,744	64.21%	3790	22.65%	1727	10.32%
Living without family	190	3.49%	3818	70.07%	1035	18.99%	406	7.45%
*p*-value	0.011		<0.001		<0.001		<0.001	
Living alone	60	2.72%	1452	65.94%	480	21.80%	210	9.54%
Not living alone	601	3.01%	13,110	65.62%	4345	21.75%	1923	9.63%
*p*-value	0.458		0.763		0.957		0.894	

NOTE: Statistically significant relationships are shown in blue.

**Table 4:** Analysis of the frequency of occurrence of a BMI category in the regions of Spain.

Regions	Under Weight (UW) BMI < 18.5 kg/m^2^		Normal Weight (NW) 18.5 kg/m^2^ <BMI < 25 kg/m^2^		Over Weight (OW) 25 kg/m^2^ < BMI < 30 kg/m^2^		Obesity (O) BMI > 30 kg/m^2^	
	N	%	N	%	N	%	N	%
Andalucía	75	2.87%	1695	64.77%	564	21.55%	283	10.81%
Aragón	18	2.76%	446	68.40%	124	19.02%	64	9.82%
Cantabria	3	1.38%	146	67.28%	48	22.12%	20	9.22%
Castilla y León	37	2.90%	838	65.67%	277	21.71%	124	9.72%
Castilla-La Mancha	27	3.70%	459	62.96%	162	22.22%	81	11.11%
Cataluña	104	2.94%	2317	65.45%	752	21.24%	367	10.37%
Ceuta y Melilla	1	3.57%	19	67.86%	5	17.86%	3	10.71%
Navarra	11	3.40%	233	71.91%	61	18.83%	19	5.86%
Comunidad Valenciana	118	3.05%	2481	64.04%	954	24.63%	321	8.29%
Comunidad de Madrid	136	3.50%	2643	68.08%	782	20.14%	321	8.27%
Extremadura	17	4.30%	226	57.22%	107	27.09%	45	11.39%
Galicia	31	2.21%	913	65.03%	313	22.29%	147	10.47%
Islas Baleares	8	1.64%	344	70.49%	98	20.08%	38	7.79%
Canarias	14	2.20%	387	60.75%	144	22.61%	92	14.44%
La Rioja	5	4.35%	75	65.22%	24	20.87%	11	9.57%
País Vasco	27	2.92%	631	68.22%	183	19.78%	84	9.08%
Asturias	20	3.03%	411	62.18%	155	23.45%	75	11.35%
Región de Murcia	9	2.16%	298	71.46%	72	17.27%	38	9.11%

**Table 5:** Sociodemographic characteristics of participants with obesity (N = 2133).

		Mean (SD) or N (%)
Sex	Male	424 (19.9%)
	Female	1709 (80.1%)
Age in years	Age total (years)	38.6 (11.3)
	Age male (years)	42.0 (12.7)
	Age female (years)	37.7 (10.8)
Education level	Basic Education	894 (41.9%)
	Higher Education	1239 (58.1%)
Income level	Low	1068 (53.2%)
	Medium-high	941 (46.8%)
Municipality	<2000	127 (6.0%)
	2000–10,000	374 (17.5%)
	>10,000	1632 (76.5%)
Living arrangement	Living alone	210 (9.8%)
	Not living alone	1923 (90.2%)
Family life	Living with family	1727 (81.0%)
	Living without family	406 (19.0%)

**Table 6:** Nutritional, social, and lifestyle variables in respondents with obesity regarding sociodemographic variables.

**Numerical Variable**	**Male**	**Female**	***p*-Value ^$^**	**Young**	**Adults**	***p*-Value ^$^**
	**Mean (SD)**	**Mean (SD)**		**Mean (SD)**	**Mean (SD)**	
IASE	50.7 (10.8)	51.5 (10.8)	0.196	49.8 (11.1)	52.0 (10.3)	<0.001
Fried food	2.50 (0.85)	2.34 (0.84)	0.001	2.54 (0.86)	2.31 (0.83)	<0.001
Fast food	2.53 (0.83)	2.55 (0.78)	0.601	2.75 (0.74)	2.47 (0.80)	<0.001
Ultra-processed food	2.40 (1.01)	2.59 (1.00)	<0.001	2.74 (0.96)	2.48 (1.01)	<0.001
Fish	1.92 (0.54)	1.80 (0.52)	<0.001	1.70 (0.52)	1.87 (0.52)	<0.001
Sport	128 (169)	81.4 (124)	<0.001	102 (144)	86.4 (132)	0.028
Sleeping hours	2.31 (0.80)	2.33 (0.80)	0.553	2.49 (0.80)	2.27 (0.79)	<0.001
Getting up rested	2.55 (0.59)	2.41 (0.59)	<0.001	2.44 (0.59)	2.43 (0.59)	0.879
Sleep quality	3.32 (1.07)	3.05 (1.09)	<0.001	3.22 (1.06)	3.06 (1.10)	0.005
Obesophobia	3.91 (1.30)	4.77 (1.16)	<0.001	4.67 (1.23)	4.57 (1.24)	0.091
No control over food intake	3.22 (1.33)	3.84 (1.34)	<0.001	3.74 (1.36)	3.71 (1.36)	0.744
Body image	3.64 (1.37)	4.55 (1.21)	<0.001	4.53 (1.29)	4.30 (1.29)	<0.001
	**Low Education**	**High Education**	***p*-Value ^$^**	**Low Income**	**Medium-High Income**	***p*-Value ^$^**
	**Mean (SD)**	**Mean (SD)**		**Mean (SD)**	**Mean (SD)**	
IASE	50.1 (11.0)	52.3 (10.1)	<0.001	50.3 (11.0)	52.7 (9.92)	<0.001
Fried food	2.43 (0.87)	2.33 (0.82)	0.012	2.37 (0.85)	2.36 (0.83)	0.639
Fast food	2.57 (0.80)	2.52 (0.79)	0.159	2.57 (0.78)	2.53 (0.80)	0.350
Ultra-processed food	2.61 (1.02)	2.51 (0.99)	0.033	2.62 (1.00)	2.48 (1.01)	0.002
Fish	1.76 (0.55)	1.86 (0.50)	<0.001	1.76 (0.53)	1.89 (0.51)	<0.001
Sport	90.8 (142)	90.5 (130)	0.265	84.6 (129)	96.5 (142)	0.092
Sleeping hours	2.27 (0.84)	2.36 (0.77)	0.006	2.32 (0.82)	2.32 (0.77)	0.911
Getting up rested	2.39 (0.61)	2.47 (0.58)	<0.001	2.41 (0.60)	2.46 (0.58)	0.031
Sleep quality	3.00 (1.11)	3.18 (1.06)	<0.001	3.02 (1.10)	3.19 (1.07)	<0.001
Obesophobia	4.50 (1.31)	4.67 (1.18)	0.005	4.57 (1.24)	4.63 (1.22)	0.346
No control over food intake	3.66 (1.42)	3.76 (1.31)	0.101	3.70 (1.35)	3.74 (1.37)	0.524
Body image	4.34 (1.32)	4.39 (1.27)	0.452	4.38 (1.29)	4.35 (1.29)	0.550
	**Living with Family**	**Living Without Family**	***p*-Value ^$^**	**Living Alone**	**Not Living Alone**	***p*-Value ^$^**
	**Mean (SD)**	**Mean (SD)**		**Mean (SD)**	**Mean (SD)**	
IASE	51.7 (10.4)	50.1 (11.4)	0.032	51.6 (10.4)	49.2 (11.9)	0.016
Fried food	2.40 (0.84)	2.26 (0.84)	0.003	2.40 (0.84)	2.16 (0.81)	0.841
Fast food	0.53 (0.79)	2.61 (0.80)	0.087	2.55 (0.79)	2.54 (0.84)	0.360
Ultra-processed food	2.54 (1.01)	2.59 (0.99)	0.382	2.56 (1.00)	2.49 (1.02)	0.002
Fish	1.84 (0.53)	1.74 (0.52)	<0.001	1.83 (0.53)	1.71 (0.52)	<0.001
Sport	89.2 (134)	96.8 (143)	0.285	91.0 (135)	87.8 (139)	0.903
Sleeping hours	2.31 (0.80)	2.38 (0.79)	0.091	2.33 (0.80)	2.33 (0.81)	0.980
Getting up rested	2.43 (0.59)	2.44 (0.61)	0.721	2.43 (0.59)	2.44 (0.61)	0.852
Sleep quality	3.09 (1.09)	3.16 (1.08)	0.215	3.10 (1.09)	3.14 (1.06)	0.439
Obesophobia	4.56 (1.24)	4.78 (1.21)	<0.001	4.59 (1.23)	4.67 (1.27)	0.310
No control over food intake	3.69 (1.37)	3.83 (1.30)	0.075	3.71 (1.37)	3.80 (1.31)	0.410
Body image	4.32 (1.31)	4.57 (1.19)	0.001	4.35 (1.30)	4.50 (1.23)	0.164

^$^ Mann-Whitney U Test; NOTE: Statistically significant relationships are shown in blue.

**Figure A4:**
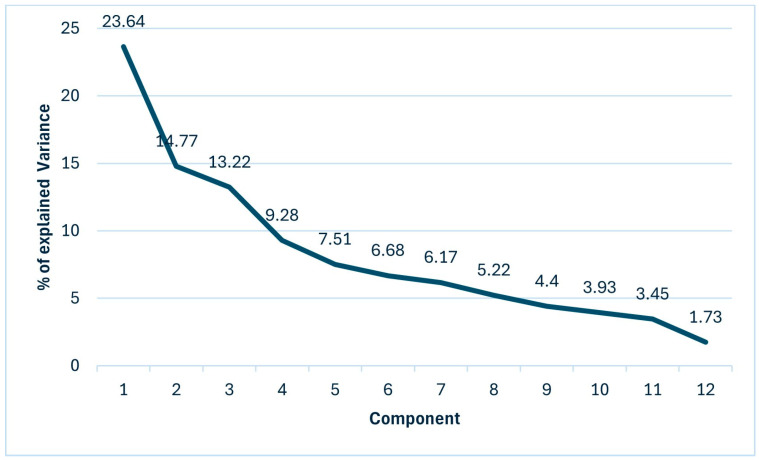
Scree plot of the PCA model.

## Data Availability

The data presented in this study are available from the corresponding author upon reasonable request.

## Appendix B

The selection of the appropriate number of principal components in the PCA is facilitated by the graph in Figure A4, which plots the percentage of variance explained along with the number of dimensions. In this study, two components were chosen, which explained 38.41% of the variance in the data, following the elbow method.
